# Exploring the Therapeutic Potential of *Artemisia* and *Salvia* Genera in Cancer, Diabetes, and Cardiovascular Diseases: A Short Review of Clinical Evidence

**DOI:** 10.3390/jcm14031028

**Published:** 2025-02-06

**Authors:** Wilson R. Tavares, Ana M. L. Seca, Maria Carmo Barreto

**Affiliations:** 1 University of the Azores, Faculty of Sciences and Technology, Centre for Ecology, Evolution and Environmental Changes (cE3c), Azorean Biodiversity Group & Global Change and Sustainability Institute (CHANGE), 9501-321 Ponta Delgada, Portugal; wilson.r.tavares@uac.pt (W.R.T.); maria.cr.barreto@uac.pt (M.C.B.); 2 Associated Laboratory for Green Chemistry (LAQV) of the Network of Chemistry and Technology (REQUIMTE), University of Aveiro, 3810-193 Aveiro, Portugal

**Keywords:** *Salvia*, *Artemisia*, clinical trial, metabolic disorders, anticancer, cardiovascular diseases, diabetes, dyslipidemia, *Salvia miltiorrhiza* depside salt

## Abstract

Metabolic syndrome, a cluster of metabolic disorders comprising dyslipidemia, insulin resistance, elevated blood pressure, and abdominal obesity, is a silent epidemic that may lead to outcomes such as cardiovascular disease, diabetes, and cancer. Due to the increase in the prevalence of these pathologies, the search for better treatments and more efficient drugs is imperative. Species of *Artemisia* and *Salvia* genera are excellent examples of noteworthy sources of bioactive products with health applications, their therapeutic properties being well known both in popular medicine and in the scientific community. There are reports of plant extracts or compounds from species belonging to either of these genera, which were able to combat cancer, diabetes, and cardiovascular pathologies. For instance, dihydroartemisinin (analog of artemisin extracted from *Artemisia annua* L.) can reduce tumor markers p53 and Ki-67 expression levels, leading to a reduction in tumor proliferation. *Salvia officinalis* L. has antihyperglycemic and lipid profile-improving effects since it decreases total cholesterol, glycosylated hemoglobin, fasting glucose, low-density lipoprotein cholesterol, and triglyceride levels while increasing high-density lipoprotein cholesterol levels. Clinical trials using mixtures (dried powdered plants or extracts) of known medicinal plants are recurrent in published works, in contrast with the scarce clinical trial studies with isolated compounds. *Salvia miltiorrhiza* Bunge. was by far the most targeted plant in the clinical trials analyzed here. Regarding clinical trials concerning *Artemisia*, there are more studies aiming to see its effect on diabetes, but the studies about cancer are more advanced. This review aims to give a critical summary of the most interesting and promising results from clinical trials. The abundance of studies with limited statistically significant clinical evidence hinders progress in clinical therapy. This situation demands far greater rigor from the scientific community, researchers, regulatory agencies, editors, and reviewers in conducting and publishing clinical studies.

## 1. Introduction

Metabolic disorders leading to what is currently known as metabolic syndrome (MS) are on the rise because of several factors such as increased sedentarism and caloric excess intake [1], sleep disturbances [2], genetic factors [3] and modifications in the composition and function of gut microbiota [4]. MS is a condition encompassing several metabolic dysregulations, namely insulin resistance, dyslipidemia, central obesity, and hypertension. Although the definition of this condition has evolved since it was initially proposed, nowadays, it is diagnosed if at least three out of five symptoms coexist in the patient: i.e., central obesity, hyperglycemia, increased triglyceridemia, low high-density lipoprotein (HDL) cholesterol, and elevated blood pressure [1]. Although each of these disorders by itself is correlated with an increased risk of type 2 diabetes and cardiovascular disease development, the risk is vastly increased by having a concurrence of these metabolic dysregulations in a way that far exceeds the sum of the individual risks caused by each condition per se [5]. MS, and even the presence of any of the metabolic disorders that characterize it, has also been associated with an increased risk of colorectal, pancreatic, bladder, and postmenopausal breast cancers [6].

After the onset of MS, insulin resistance, chronic inflammation, and changes in the levels of adipokines are most likely some of the mechanisms involved in the progression of these metabolic dysregulations [1]. MS also increases the risk of developing cancer, namely by being correlated with an increase in xanthine oxidoreductase activity, causing higher formations of reactive oxygen species (ROS) and uric acid, which in turn causes an upregulation of the renin/angiotensin pathway and an increase in cyclooxygenase-2 (COX-2) expression [7]; therefore, increased xanthine oxidoreductase activity is correlated with MS and cancer development. The high incidence of cancer in patients with MS is also related to the overactivation of the hypothalamus–pituitary–adrenal axis, leading to hypercortisolemia and immune impairment, which in turn favors chronic inflammation and tumorigenesis [8].

Traditional herbal medicine is a practice as old as humanity [9] and continues to be widely practiced around the world [10] to treat a wide range of ailments, including the treatment of some metabolic dysregulations associated with MS [11,12]. In this regard, several therapeutic effects of *Artemisia* [13,14,15] and *Salvia* [16,17,18,19] species have been reported. For instance, *Artemisia absinthium* L., in addition to its vast use in the cosmetic and food industries [20], is traditionally used in worldwide folk medicine to alleviate digestive discomforts and gastrointestinal problems [21]. In the Eastern Cape region of South Africa, inhaling vapors of boiled leaves of *Artemisia afra* Jacq. ex Willd. is a common practice to treat various respiratory ailments, while drinking an infusion of roots and leaves of the plant is used for the treatment of diabetes [22]. In China, a daily infusion of 4.5 g to 9 g of dried *Artemisia annua* L. is suggested for the treatment of malaria and fever [23]. *Artemisia annua* is also traditionally used as a remedy in the form of aqueous extracts, infusions, and tinctures from the dried plant for bleeding wounds, hemorrhoids, infections, and even tumors [24]. *Artemisia scoparia* Waldst. and Kit. is another medicinal plant that has a long-recorded therapeutic use in Asia and Central Europe, being used to treat cardiovascular and inflammatory conditions, diabetes, and hyperglycemia, as well as gallbladder, liver, and digestive problems [13]. While most preparations (e.g., decoctions or infusions) come from the aerial parts of *A. scoparia*, roots can also be used, and in Iran, the flower is preferred [25]. Furthermore, dried aerial parts of *Artemisia capillaris* Thunb. can be added sometimes to the preparations to increase their medicinal properties [13]. Many other traditional uses of *Artemisia* species are reported across the literature, with Nurlybekova and colleagues [26] presenting a very good and recent review of the topic that is recommended for further reading.

*Salvia* species uses in folk medicine are also various and noteworthy [27]. For example, Southern Africa is the home of traditional medicine involving preparations by decoctions of leaves from *Salvia africana* L., *Salvia chamelaeagnea* Berg., and *Salvia dentata* Aiton, used to treat stomach complaints, influenza, colds, and coughs [28]. In traditional Chinese medicine, *Salvia miltiorrhiza* Bunge is a popular plant used to treat cardiovascular and renal disorders [29]. Native to Mexico and Ecuador, *Salvia hispanica* L. (commonly known as ‘*chia*’) has been used since ancient times for its high content of protein and its medicinally associated properties such as blood sugar control and weight management, as well as bone, cardiovascular, and digestive health [30]. The essential oils and water extracts of *Salvia fruticosa* Mill. (syn. *Salvia libanotica* Boiss. and Gaill.), in addition to being externally applied to heal fractured bones or burns, they are also used as infusions, or their steams are inhaled in order to treat cardiovascular problems, headaches, indigestion, and abdominal pain [31]. *Salvia officinalis* L. and *Salvia officinalis* subsp*. lavandulifolia* (Vahl) Gams (syn. *Salvia lavandulifolia* Vahl) are reported in European herbal encyclopedias as having a memory-enhancing effect [32,33]. In addition, *S. officinalis* is widely used worldwide to treat colds, bronchial infections, and coughs [34].

**Figure 1:**
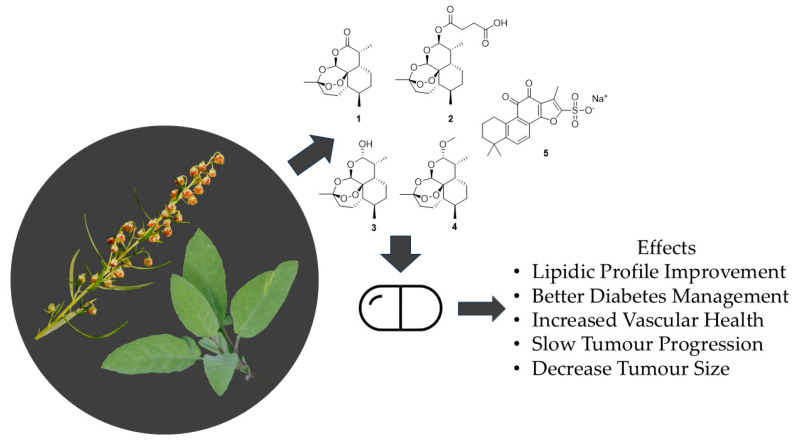
*Artemisia* and *Salvia* have various phytochemicals that provide beneficial effects against cancer, diabetes, and cardiovascular disease, e.g., 1—Artemisinin; 2—Artesunate; 3—Dihydroartemisinin; 4—Artemether; 5—Sodium tanshinone IIA sulfate.

In addition to the effects reported in traditional medicine, studies have started to unveil the biological processes behind the beneficial health properties (Figure 1) of both plant genera. For instance, in a study with obese rats, *S. miltiorrhiza* hydroethanolic root extract significantly reduced glucose levels, adipocyte vacuolation, body fat index, weight, and lipid profile. These changes were associated with better lipid metabolism and improved gut integrity, as well as increased levels of lipid-related factors such as hormone-sensitive lipase (HSL), 3′-5′-Cyclic adenosine monophosphate (cAMP), and AMP-dependent protein kinase (PKA) in the liver and adipose tissue [35]. In similar studies, *Artemisia capillaris* Thunb. extracts were used to treat high-fat diet rats, leading to their decreased body weight and levels of triglycerides, low-density lipoprotein (LDL) cholesterol, and total cholesterol, with the effects being mediated by the downregulation of fatty acid synthase genes through the miR-122 pathway [36,37].

It was found that *S. officinalis* had DNA-protective effects and chemo-preventive properties over colorectal cancer in an in vivo study [38]. In addition to a decrease in tumor marker Ki67 levels, the results also demonstrate that *S. officinalis* treatment prevented DNA damage by azoxymethane on lymphocytes (by 65%) and colonocytes (by 20%) and by H_2_O_2_ on colonocytes (by 28%), while inhibiting azoxymethane-induced the proliferation of mucosal cells by 30.5%. Phytochemicals from *A. annua*, such as artemisinin analogs, i.e., artesunate and dihydroartemisinin (Figure 1), were found to have anticancer properties against pancreatic cancer [39]. Acting through the NF-κB pathway [40] and altering mitochondrial membrane potential [41], tumor progression was slowed, and tumor mass and volume decreased with no body weight loss in pancreatic cancer xenograft model rats.

Since higher ROS accumulation can induce gene mutations and promote carcinogenesis, substances with high antioxidant activity can lower oxidative stress and impair cancer development [42]. Thus, another explanation for the anticancer effects of *Artemisia* and *Salvia* may reside in their high content of compounds with antioxidant properties that improve the activity of catalase, glutathione peroxidase, glutathione reductase, and superoxide dismutase, which lead to lower production of ROS [43,44].

Considering the threat that metabolic disorders present in terms of morbidity and mortality worldwide, it is important to discover drugs that can act on targets related to the metabolic pathways involved in these multifactorial diseases, in addition to promoting changes in lifestyle, increased physical activity, and dietary corrections. It is, therefore, crucial to discover compounds that are active against enzymes, receptors, and pathways correlated with the development of these multifactorial diseases and/or with their consequences. In this context, there are a high number of reports of therapeutic effects of *Artemisia* and *Salvia* species related to MS, cancer, inflammation and other affections linked with these diseases. In vitro and in vivo studies will not be the scope of this review since they are the first steps in the drug development process but do not reflect the true potential and limits of a drug in coping with a specific metabolic disorder.

Given the increasing prevalence of metabolic syndrome and its multifaceted complications, a comprehensive evaluation of available clinical evidence is essential to assess the efficacy and safety of these botanical resources. This critical review aims to consolidate and analyze the most relevant clinical trials, and case reports that explore the therapeutic applications of bioactive compounds and extracts derived from species of the *Artemisia* and *Salvia* genera in the treatment of metabolic syndrome-associated pathologies, including cancer, diabetes, and cardiovascular diseases. This review places particular emphasis on the pharmacological effects of both isolated secondary metabolites and complex plant preparations, highlighting their mechanisms of action, clinical outcomes, and potential limitations.

Dissimilar to previous studies that primarily focus on in vitro and in vivo research, this review systematically compiles and analyzes clinical trials, emphasizing their methodological limitations and the challenges in translating preclinical findings into clinical practice. It distinguishes itself by critically evaluating trial methodologies, the availability of standardized extracts and pure compounds, and the reliability of clinical data supporting the therapeutic potential of these botanical species. By identifying key research gaps, this paper highlights the need for well-designed clinical studies to validate the efficacy and safety of plant-derived treatments, offering a valuable resource for researchers and clinicians advancing phytotherapy-based interventions for metabolic syndrome and related diseases.

## 2. Clinical Trials and Case Reports

Regarding the action of *Artemisia* and *Salvia* extracts and their compounds described in the literature [13,14,15,16,17,18,19], it shows that they hold significant pharmaceutical potential, with some already in clinical trial stages. In fact, a swift search on ClinicalTrials.gov [45] found a total of 52 clinical trials testing *Artemisia* (18 results) and *Salvia* (34 results) that were relevant to the theme of this review.

The current interest and importance of this topic are highlighted by the fact that, from those 50 clinical trials, three are scheduled to start shortly (two *Artemisia*-related and one *Salvia*-related), and two (*Artemisia*-related) are active and ongoing. From the 24 clinical trials already completed (Table 1) (nine *Artemisia*-related and 15 *Salvia*-related), only two (both *Artemisia*-related) had their results published. In addition, 23 clinical trials were terminated or their progress status is unknown. In order to have a better overview of this topic with growing interest, more data were obtained across the internet, and it was possible to find case reports and other clinical trials that were not listed on the ClinicalTrials database.

The studies reviewed here are divided according to the plants from which the tested substances in the clinical trials originate, as well as the different diseases that are addressed in each one.

### 2.1. Artemisia

Starting with *Artemisia* studies, the following paragraphs discuss the promising pharmaceutical properties of this plant genus against cancer and diabetes, the only diseases that fit into the scope of this article with published case reports and clinical trial results. All information in this section is summarized in Table 2.

#### 2.1.1. Cancer

**Figure 2:**
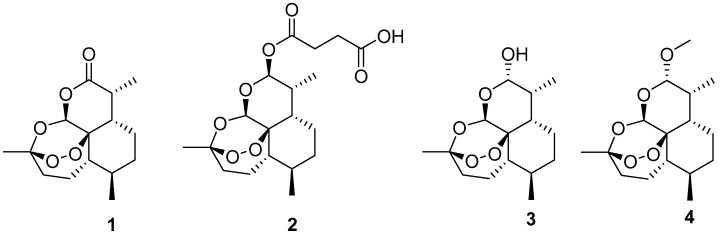
Compounds used in *Artemisia*-related clinical trials. 1—Artemisinin; 2—Artesunate; 3—Dihydroartemisinin; 4—Artemether.

Cancer is a complex, multifaceted, and problematic disease with substantial incidence all around the globe that still lacks a precise cure [46], with punctual solutions involving long and complicated treatments with unpleasant side effects and unpredictable outcomes [47]. Thus, the search for better anticancer medicines is always in high demand. Since there are no published works regarding the use of *Artemisia* genus extracts for cancer treatment, the presented clinical trials in this section were carried out with compounds present in the genus and with their analogs. More specifically, artemisinin (1) (extracted from *A. annua*) and its analogs artesunate (2), dihydroartemisinin (3), and artemether (4) (Figure 2). Although not all cancers presented here are related to obesity, these studies were included to demonstrate the anticancer potential of this plant genus.

Artesunate (a drug usually used for the treatment of malaria) was found to be effective in treating advanced non-small cell lung cancer when in combination with the standard treatment of vinorelbine and cisplatin [48]. Briefly, during two cycles of 21 days each (eight days of consecutive treatment followed by rest), 60 patients of the control group received only the standard treatment (i.e., intravenous injection of 25 mg/m^2^ of vinorelbine once-a-day at the 1st and 8th days and intravenous drip of 25 mg/m^2^ of cisplatin once-a-day from the 2nd to 4th days), while other 60 patients received, in addition to the standard treatment, a daily intravenous artesunate injection (120 mg) for the first eight days. The results of the clinical trial point out a significantly longer time (*p* < 0.05) for disease progression in the artesunate injection group (24 weeks) than in that of the control group (20 weeks). In addition, artesunate-treated patients had a disease control rate of 88.2%, significantly higher (*p* < 0.05) than that of the control group (72.7%). Taking these results into account, the authors of the work suggest that artesunate can be combined with existing chemotherapeutic agents without extra side effects to delay the time of progression of the disease and increase the survival rate of patients [48].

More recently, a randomized, placebo-controlled, single-center, double-masked clinical trial also demonstrated the anticancer properties of artesunate [49]. After diagnosis and before surgery, 23 colorectal carcinoma subjects were given either 14 daily doses of placebo (n = 11) or oral artesunate (200 mg; n = 12). Clinical survival, immunohistochemical assessment in terms of the expression level of several tumor markers (i.e., CD31, c-MYC, EGFR, Ki-67, p53, and VEGF) and the percentage of tumor cells in apoptosis (significant if >7% measured by the TUNEL assay staining) were the parameters measured in the study. The results point out the antiproliferative properties of artesunate since, using Bayesian analysis, it was found that artesunate treatment had a probability factor of 0.89 of reducing Ki-67 expression. The upregulation of Ki-67 is connected with a worse prognosis in colorectal cancer [50]; thus, the low expression of this marker is a good anticancer sign. Furthermore, after a follow-up of 42 months, resurgent colorectal carcinoma was present in six placebo-treated patients, whilst it was only present in one artesunate-treated patient [49].

In a small-scale pilot clinical trial [51], ten patients with advanced cervical cancer were treated for 28 days with an oral dihydroartemisinin dose (100 mg/day in the 1st week, 200 mg/day in the following weeks), with all patients showing fast clinical improvement and tumor remission during and after the treatment period. With no observable severe toxicity and the occurrence of some tolerable adverse events (abdominal pain and headache), vaginal discharge and pain (clinical symptoms of the disease) disappeared in a median time of seven days. Immunohistochemistry revealed that the observed effects in this clinical trial were associated with a decreased expression level of relevant tumor markers p53 and Ki-67, indicating that tumor proliferation was slowed down. Tumour relapsed in six patients after an average of six months, with four of them being treated for a second time with the same protocol and achieving, again, clinical remission after 28 days of treatment. The results obtained by this pilot clinical trial [51] are very encouraging, paving the way for larger, prolonged, and randomized controlled trials.

In addition to the clinical trials mentioned above, some case reports were also published with promising conclusions in terms of the use of artemisinin and its analogs against cancer. The first case reports of the successful use of artemisinin or its analogs for cancer treatment were published by Rowen in 2002 [52], where patients experienced either cancer regression at the end of treatment or were cancer-free. A patient (47-year-old) with non-Hodgkin’s lymphoma had a 4.5 cm lymph node tumor and received intramuscular injections (60 mg of artesunate) for 14 days. One month later, the mass was completely absent and stayed that way for at least the time the article was written (6 months later). Another 47-year-old patient received orally a combination of artemisinin and artesunate (300 mg) two times a day to treat her metastatic stage 4 breast cancer. Six months later into treatment, the report stated that she had no symptoms whatsoever, and the regressed mass was determined to be only scar tissue. One more case of a woman in her 40s with metastatic breast cancer was also reported in the same article. The patient received radiation therapy prior to the oral artemisinin treatment (posology and concentration not specified). The patient reported improvement in symptoms and general well-being after two weeks of starting treatment, being declared cancer-free after four months. Artemisinin was also used in another case to treat persistent multiple skin cancer in an 81-year-old patient. Twice daily topical application of artemisinin (unknown concentration in 50% DMSO) caused the principal tumor to fall off within 5 days and other smaller skin cancers to regress. The last case addressed here is the one of an 83-year-old female patient who saw her non-resectable, non-small cell lung carcinoma shrink and disappear throughout 4 months of oral artemisinin treatment (500 mg, two times a day) via nebulizer [52].

Another example of artesunate in cancer treatment was reported by Singh & Verma [53]. A 72-year-old man diagnosed with laryngeal squamous cell carcinoma saw his tumor reduced by 70% after nine months of artesunate treatment, i.e., 15 days of intramuscular injections (60 mg), followed by oral intake via tablet (50 mg) afterward. With no visible adverse side effects, the patient’s quality of life improved and was extended by eight months when compared with other cases of this specific cancer type.

Another artemisinin analog, i.e., artemether, was used to treat a pituitary macroadenoma in a 75-year-old male patient [54]. A daily oral dose of 40 mg improved the patient’s vision after 15 days of starting the treatment. After 12 months of treatment, tumor density decreased, and normal vision was reported by the patient without any complaint of side effects.

Although there are no reports of clinical trials using *Artemisia* extracts, a case report [55] used capsules containing 50 mg of *A. annua* extract. The pills were taken orally five times a day for nine months by an 80-year-old patient with metastasized and progressive prostate cancer. Upon one month of treatment, the tumor marker PSA (prostate-specific antigen) level in blood intensely dropped from 580.3 µg/L to 0.98 µg/L. In addition, several lipomas present at the inner side of the thighs regressed, as well as a decrease in prostate carcinoma-related symptoms. Unfortunately, despite the remarkable initial sensitivity and the continuation of treatment, the tumor developed resistance after 7 months. PSA levels increased, and tumor resurgence with metastasis followed. Nevertheless, the authors suggest that these results draw the base for long-term treatment with *A. annua* capsules.

#### 2.1.2. Diabetes

The incidence of diabetes and pre-diabetes, as well as the prevalence of cardiovascular disease, are rapidly growing worldwide [56]. In fact, when compared with individuals with normal glycemic levels, 10% of individuals with pre-diabetes or diabetes have a major risk of developing cardiovascular diseases within 10 years [57]. With that in mind, various studies have been taking place to find alternatives to the already existing therapies that rely on synthetic drugs to improve glycemic control in diabetic and pre-diabetic patients.

A study was carried out as an initial assessment of the dose/response of an *Artemisia princeps* Pamp. ethanolic extract on hyperglycemic patients [58]. In this randomized, placebo-controlled and double-masked clinical trial, 80 hyperglycemic subjects (values of 100 to 150 mg/dL for fasting blood glucose; 51 males and 29 females) were arbitrarily assigned to four groups of twenty persons, i.e., the placebo (lactose, 2000 mg/day), the positive control (a compound found in carob that displays antidiabetic activity, pinitol, 1140 mg/day), the low-dose extract (2000 mg/day) and the high-dose extract (4000 mg/day) group. After 8 weeks of supplementation, when compared with the baseline values, fasting blood glucose and glycosylated hemoglobin levels dropped significantly in both extract groups, while no significant variations were reported in the placebo or the positive control groups. The low-dose extract treatment caused a decrease in fasting blood glucose from 123.27 ± 4.66 mg/dL to 116.93 ± 5.00 mg/dL (*p* < 0.05), while the high-dose extract treatment caused a decrease from 125.50 ± 4.43 mg/dL to 115.13 ± 4.50 mg/dL (*p* < 0.01). Regarding glycosylated hemoglobin levels in the low-dose and high-dose extract groups, they dropped from 5.70 ± 0.16% to 5.34 ± 0.15% (*p* < 0.05) and from 5.85 ± 0.18% to 5.39 ± 0.22% (*p* < 0.05), respectively. Based on the observed results, and since there were no secondary effects, the authors of this clinical trial conclude that supplementation with *A. princeps* is well tolerated up to 4 g a day for at least 28 days and can be used to improve glycemic control in humans, but suggest that larger clinical trials should take place to confirm the efficacy of the tested treatment [58]. It should be noted that this study was carried out with an unbalanced number of gender participants, which may influence the obtained results since the sample is not homogeneous.

Cho and colleagues [59] conducted a double-masked, randomized, placebo-controlled clinical trial to assess the antidiabetic properties of an *A. princeps* extract on subjects diagnosed with pre-diabetes (fasting blood glucose values of 100 mg/dL to 125 mg/dL, while normal values are below 100 mg/dL [60]). In addition, all individuals presented glycosylated hemoglobin levels symptomatic of long-term moderate hyperglycemia, i.e., from 5.5% to 6.3% (individuals with levels between 6% to 6.4% have an increased risk of diabetes development, ≥6.5% are diagnosed as diabetic [60]). Briefly, 99 persons (42 males and 57 females) were arbitrarily assigned into three different groups with the same number of people, i.e., the positive-control (1140 mg/day of pinitol), the placebo (starch, 2000 mg/day), and the *A. princeps* extract (3000 mg/day) groups, and given their corresponding treatment for a course of nine weeks. After that treatment period, the extract group presented a decrease in fasting blood glucose levels of 16.51 ± 2.78% (*p* < 0.05), while the pinitol and the placebo groups presented, respectively, decreases of 7.28 ± 1.95% (*p* < 0.05) and 2.21 ± 2.91% (*p* < 0.05). In addition, the glycosylated hemoglobin levels only decreased in the extract group (from 6.28 ± 0.13% to 5.63 ± 0.10%, *p* < 0.05), while they increased in the pinitol (from 5.54 ± 0.18% to 6.05 ± 0.18%, *p* < 0.05) and placebo (from 6.19 ± 0.28% to 6.51 ± 0.22%, *p* < 0.05) groups. Furthermore, no adverse effects were described. Unfortunately, as mentioned by the authors of the study [59], the compounds responsible for the antidiabetic effects observed in this clinical trial were not identified. This is a point that should be studied, since it is essential for understanding the mechanism of action behind the obtained results.

A water extract of *Artemisia dracunculus* L. (aerial part) was used in a randomized, double-masked, cross-over small clinical trial to investigate its possible acute effects on insulin and glucose levels in response to an oral glucose tolerance test [61]. After a 10 h overnight fast and 15 min prior to an oral glucose tolerance test (75 g of dextrose), 12 young (24.8 ± 5.3 years), healthy and non-diabetic men were randomly assigned to consumed either a capsule containing 2 g of placebo (cellulose) or 2 g of aqueous extract of *A. dracunculus*. Blood samples were taken before the ingestion of the capsules and every 15 min after ingestion of the dextrose, until the 75 min mark. One week later, the subjects repeated the test but were provided with the capsule that they did not ingest the first time. The conclusion of this study is that the acute ingestion of a water extract of *A. dracunculus* results in a minor drop in insulin and glucose blood levels, although both parameters failed to reach statistical significance. It is pointed out by the authors that the low response of the tested extract could be due to subjects being healthy and, therefore, not needing additional support to help maintain ideal glucose and insulin levels; thus, further studies, including a bigger sample of pre-diabetic or diabetic people, are needed [61].

The effect of *A. dracunculus* ethanolic extract was also studied in a more recent clinical trial [62]. Twenty-four patients with impaired glucose tolerance (20 females and 4 males) participated in a randomized, double-masked, placebo-controlled study to evaluate the outcome of *A. dracunculus* on insulin secretion, insulin sensitivity, and glycemic control. After randomization into two groups with the same number of people, each subject was prescribed 90 days of capsules containing the placebo (2 g/day of calcined magnesia) or ethanolic extract of *A. dracunculus* leaves (2 g/day). By the end of treatment, no significant differences were observed after placebo administration, but circulating glucose, insulin, and insulin secretion presented significant decreases (*p* < 0.05) in the patients that took *A. dracunculus* capsules, indicating an improvement in insulin sensitivity and glycemic control. Furthermore, it should be pointed out that the non-placebo group also presented a significant rise (*p* < 0.05) in HDL cholesterol levels. Nonetheless, the authors [62] point out that a limitation of their study could be the fact that it was conducted with mostly females; thus, future studies should aim for a more homogenous study population with an equal representation of both sexes since insulin sensitivity can be influenced by hormonal factors.

Sun and colleagues [63] carried out a 10-week randomized, double-masked, and placebo-controlled trial designed to ascertain the effects of *A. scoparia* extract (a 99.9% pure extract certified) on insulin sensitivity and lipid profiles of singleton pregnant women diagnosed with gestational diabetes mellitus at their second trimester (13 weeks of pregnancy). The 144 participants were, at random, equally distributed to the placebo group or the extract group and took, according to their group, a daily dosage of 400 mg of placebo or extract in the form of tablets. Fasting blood samples were collected from all participants at weeks zero and ten. After the treatment period, and compared with the placebo group, the serum insulin and fasting plasma glucose levels were significantly reduced in the extract group by 13.64% and 9.58%, respectively. In addition, circulating adiponectin levels were significantly increased by 73.08% in the extract group while increasing by only 10.14% in the placebo group. This is a very promising result since reduced levels of adiponectin are linked with the further development of gestational diabetes mellitus [64]. Taking this into account, the authors conclude that the observed improvement in insulin sensitivity is owed to the high levels of adiponectin and propose daily administration of *A. scoparia* extract in gestational diabetes mellitus patients in order to improve insulin and glucose metabolism [63]. Although promising results were obtained, it must be highlighted that the use of brown sugar as a placebo in a diabetic study, and with the justification of “brown sugar is identical in appearance as *Artemisia scoparia* extract”, is a strange methodological approach since it could impair the general outcome.

Antihyperlipidemic and hypoglycemic effects of three plants separately, *Citrullus colocynthis* (L.) Schrad., *Gymnema sylvestre* (Retz.) R.Br. ex Sm., and *A. absinthium* were assessed in a small, single-masked, randomized placebo-controlled clinical trial in type II diabetic patients [65]. Thirty-two male and female individuals with type II diabetes were randomly assigned to the test groups and the placebo group (eight persons each). In relation to the group that receives *A. absinthium*, for the course of 30 days, two capsules, each containing 0.5 g of *A. absinthium* (leaves) powder, were provided to the test group, while the placebo group was provided with two capsules containing each 0.5 g of placebo (not specified), followed by 10 days wash period where no dose was given to either group. During the 40-day experimental period, subjects were allowed to use their routine oral hypoglycemic drugs and/or routine dietetic food as prescribed by their doctors. From day zero to day forty, blood samples were collected in fasting condition from each individual every 10 days. Antihyperlipidemic effects were not significant (*p* < 0.05), but the average fasting serum glucose level decreased gradually for 30 days from 211 ± 57 mg/dL to 143 ± 30 mg/dL and was 191 ± 26 mg/dL 10 days after stopping the treatment of the tested supplementation, showing a significant lowering effect (*p* < 0.05) when compared with the placebo group. The authors [65] suggest that the addition of 1 g/day of *A. absinthium* could work synergistically with other medications to help achieve better glycemic control.

Three years later, Hassan and colleagues [66] published a very similar study using the same plants and methods, with the obtained results being almost identical to the ones described above, with insignificant differences. For example, compared with the original clinical trial [65], this study [66] presents the same values of fasting serum glucose level at day zero for all the groups with just only one unit, randomly, up or down in each group, but with the same standard error. The same is observed for the obtained results after the 40-day treatment period. In fact, even the geographical area where both studies were carried out is the same (i.e., Lakki Marwat district). It could be that we are facing a second attempt at the clinical trial to confirm the results of the first one, but none of the authors are the same as the initial study, and the work by Li et al. [65] is never mentioned. This situation casts many doubts concerning the veracity of the obtained results.

**Table 2:** Summary of clinical studies and their results involving *Artemisia* species.

Disease	*Artemisia* Species	Compound/Drug/Extract	Type of Study	Number of Participants	Intervention	Main Outcomes	Reference
Cancer	*Artemisia annua* L.	Artemisinin	Case Report	1 (Metastatic stage 4 breast cancer)	Combinatory Treatment (6 months): 300 mg Artemisinin, orally 2×/day + Artesunate (dose not specified), intravenous injection 1×/day	↓ Tumor mass Only scar tissue present	[52]
				1 (Metastatic breast cancer)	Oral artemisinin treatment (posology and concentration not specified)	Cancer free after 4 months	
				1 (Persistent multiple skin cancer)	5 Days Treatment: Artemisinin (unknown concentration in 50% DMSO), topical application 2×/day	↓ Tumors mass	
				1 (Non-resectable non-small cell lung carcinoma)	4 Months Treatment: 500 mg of Artemisinin, orally 2×/day	↓ Tumors mass	
		Artemether ^1^	Case Report	1 Pituitary (macroadenoma)	12 Months Treatment: 40 mg of Artemether, orally 1×/day	↓ Tumors density Normal vision restored	[54]
		Artesunate ^1^	Randomized and Controlled Clinical Trial	120 (Advanced non-small cell lung cancer)	2 Treatments (42 days, 2 cycles of 21 days each with 8 days of consecutive treatment followed by rest): Control Group (n = 60) = 25 mg/m2 of Vinorelbine, intravenous injection 1×/day (1st and 8th days) + 25 mg/m2 of Cisplatin, intravenous drip 1×/day (2nd to 4th days) Artesunate Group (n = 60) = Same as Control Group + 120 mg of Artesunate, intravenous injection 1×/day (1st to 8th days)	Significantly (*p* < 0.05) longer time for disease progression: Control Group = 20 weeks Artesunate Group = 24 weeks Significantly (*p* < 0.05) higher disease control rate: Control Group = 72.7% Artesunate Group = 88.2%	[48]
			Randomized, Single-centre, Double-masked and Placebo-Controlled Clinical Trial	23 (Colorectal carcinoma)	2 Treatments (14 days): Placebo Group (n = 11) = Placebo (not specified), orally 1×/day Artesunate Group (n = 12) = 200 mg of Artesunate, orally 1×/day	0.89 Probability factor of artesunate ↓ Ki-67 expression Resurgence after 42 months: Placebo Group = 6 resurgent patients Artesunate Group = 1 resurgent patient	[49]
			Case Report	1 (Non-Hodgkin’s lymphoma)	14 Days Treatment: 60 mg Artesunate, intramuscular injection 1×/day	Lymph node tumour disappeared	[52]
			Case Report	1 (Laryngeal squamous cell carcinoma)	9 Months Treatment: 60 mg Artesunate, intramuscular injection 1×/day for 15 days 50 mg Artesunate, orally 1×/day from the 16th day onwards	↓ Tumor size by 70%	[53]
		Dihydroartemisinin ^1^	Open-label and Single-center Clinical Trial	10 (Advanced cervical cancer)	28 Days Treatment: 100 mg Dihydroartemisinin, orally 1×/day during 1st week 200 mg Dihydroartemisinin, orally 1×/day in the following weeks	↓ Expression levels of tumour markers p53 and Ki-67 ↓ Tumor proliferation	[51]
		Extract (Not specified)	Case Report	1 (Metastasized and progressive prostate cancer)	9 Months Treatment: Capsule (50 mg *A. annua* extract) orally, 5×/day	↓ Tumor marker Prostate-specific antigen level, in 1 month, from 580.3 µg/L to 0.98 µg/L ↓ Tumors mass	[55]
Diabetes	*Artemisia absinthium* L.	Herbal Drug (*A. absinthium* Leaves Powder; other plants used in the study: *Citrullus colocynthis* (L.) Schrad. and *Gymnema sylvestre* (Retz.) R.Br. ex Sm.)	Randomized, Blind, and Placebo-controlled Clinical Trial	32 (Type II diabetic)	4 Treatments (30 days + 10 days wash period): Placebo Group (n = 8) = 1000 mg/day of Placebo (not specified), orally 2 capsules (500 mg each) *A. absinthium* Group (n = 8) = 1000 mg/day of *A. absinthium* powder, orally 2 capsules (500 mg each) *C. colocynthis* Group (n = 8) = 1000 mg/day of *C. colocynthis* powder, orally 2 capsules (500 mg each) *G. sylvestre* Group (n = 8) = 1000 mg/day of *G. sylvestre* powder, orally 2 capsules (500 mg each)	Steadily ↓ average fasting serum glucose level in *A. absinthium* Group, from 211 ± 57 mg/dL to 143 ± 30 mg/dL Significant (*p* < 0.05) ↓ of average fasting serum glucose level 10 days after stopping treatment, *A. absinthium* Group = 191 ± 26 mg/dL	[65]
	*Artemisia dracunculus* L.	Extract (Water, Aerial Part)	Randomized, Double-masked, and Cross-over Clinical Trial	12 (Healthy and non-diabetic)	2 Treatments randomly assigned (1-week apart, treatment change): Placebo Capsule (2000 mg of Cellulose, orally 1 capsule, 1 time only) Extract Capsule (2000 mg of Extract, orally 1 capsule, 1 time only)	Slight ↓ insulin and glucose blood levels after ingestion of Extract Capsule	[61]
		Extract (Ethanolic)	Randomized, Double-masked, and Placebo-controlled Clinical Trial	24 (Impaired glucose tolerance)	2 Treatments (90 days): Placebo Group (n = 12) = 2000 mg/day of Calcined magnesia, orally 4 capsules (500 mg each) Extract Group (n = 12) = 2000 mg/day of Extract, orally 4 capsules (500 mg each)	Significant (*p* < 0.05) ↓ circulating glucose, insulin, and insulin secretion levels in Extract Group Improvement in insulin sensitivity and glycemic control in Extract Group Significant (*p* < 0.05) HDL cholesterol levels in Extract Group	[62]
	*Artemisia princeps* Pamp.	Extract (Ethanolic)	Double-masked, Randomized and Placebo-controlled Clinical Trial	80 (Hyperglycemic)	4 Treatments (8 weeks): Placebo Group (n = 20) = 2000 mg/day of Lactose, orally 4 capsules (500 mg each) Positive Control Group (n = 20) = 1140 mg/day of Pinitol, orally 4 capsules (285 mg each) Low-dose Group (n = 20) = 2000 mg/day of Extract, orally 4 capsules (500 mg each) High-dose Group (n = 20) = 4000 mg/day of Extract, orally 8 capsules (500 mg each)	Significant (*p* < 0.05) ↓ glycosylated hemoglobin levels: Low-dose Group = from 5.70 ± 0.16% to 5.34 ± 0.15% High-dose Group = from 5.85 ± 0.18% to 5.39 ± 0.22% Fasting blood glucose levels: Low-dose Group = Significant (*p* < 0.05) ↓ from 123.27 ± 4.66 mg/dL to 116.93 ± 5.00 mg/dL High-dose Group = Significant (*p* < 0.01) ↓ from 125.50 ± 4.43 mg/dL to 115.13 ± 4.50 mg/dL	[58]
		Extract (Ethanolic)	Double-masked, Randomized and Placebo-controlled Clinical Trial	99 (Pre-diabetic)	3 Treatments (9 weeks): Placebo Group (n = 33) = 2000 mg/day of Starch, orally 1 capsule Positive Control Group (n = 33) = 1140 mg/day of Pinitol, orally 1 capsule Extract Group (n = 33) = 3000 mg/day of Extract, orally 1 capsule	Significant (*p* < 0.05) ↓ fasting blood glucose levels in Extract Group of 16.51 ± 2.78% ↓ Glycosylated hemoglobin levels only in Extract Group	[59]
	*Artemisia scoparia* Waldst. and Kit.	Extract (Not specified)	Randomized, Double-masked, and Placebo-controlled Clinical Trial	144 (Gestational diabetes mellitus)	2 Treatments (10 weeks): Placebo Group (n = 72) = 400 mg/day of Brown sugar, orally 2 capsules (200 mg each) Extract Group (n = 72) = 400 mg/day of Extract, orally 2 capsules (200 mg each)	Significant (*p* < 0.05) ↓ insulin and fasting plasma glucose levels in the Extract Group by, respectively, 13.64% and 9.58% Circulating adiponectin levels: Placebo Group = ↑ by 10.14% Extract Group = Significant (*p* < 0.05) ↑ by 73.08%	[63]

^1^ Analogue of artemisinin that is extracted from *Artemisia annua* L; ↑ = Higher or increase; ↓ = Lower or decrease; HDL = High-density lipoprotein.

### 2.2. Salvia

The following paragraphs will discuss the pharmaceutical properties of *Salvia* species extracts and their compounds. Again, only diseases that fit into the scope of this article, with published clinical trial results, will be addressed here, i.e., diabetes, dyslipidemia, and cardiovascular (angina and coronary heart disease) problems. All information in this section is summarized in Table 3.

#### 2.2.1. Diabetes

In women with polycystic ovary syndrome, there is an increased frequency of impaired glucose tolerance, insulin resistance, and development of type II diabetes mellitus [67]. Amini and colleagues [68] carried out a randomized, placebo-controlled, triple-blinded clinical trial using patients with polycystic ovary syndrome to evaluate the effect of *S. officinalis* ethanolic extract over insulin resistance indicators and anthropometric indexes. Seventy subjects were assigned either to the placebo (n = 35) or the *S. officinalis* extract (n = 35) groups. For 8 weeks, each woman took a daily capsule containing either 300 mg of corn starch (placebo) or powdered ethanolic extract of *S. officinalis*, according to their treatment group. The outcomes of this study indicate a statistically significant decrease in the body mass index of the extract group (i.e., minus 0.56 ± 0.90 kg/m^2^) (*p* = 0.001) when compared with the placebo group (i.e., plus 0.08 ± 0.38 kg/m^2^). Furthermore, compared with placebo (decrease of 1.68%), the consumption of *S. officinalis* extract significantly decreased insulin levels by 28.03% (*p* < 0.001) [68].

Several clinical studies have been conducted with the aim of finding clinical evidence of the effect of *Salvia* species extracts in adjunctive treatments. Kianbakht & Dabaghian [69] carried out a work where *S. officinalis* hydroethanolic leaf extract was used to treat patients with both type II diabetes and hyperlipidemia in a three-month randomized, placebo-controlled, parallel-group clinical trial. Two groups, each with forty patients, were made: the extract group (one capsule containing 0.5 g of *S. officinalis* extract every 8 h) and the placebo group (one capsule containing 0.5 g of toast powder every 8 h). In addition to the trial capsules, each patient was allowed to continue taking their routine medication, i.e., 0.01 g glyburide and 1 g metformin daily. Compared with the placebo group, the results of the study showed that *S. officinalis* treatment had a lipid profile and antihyperglycemic improving effects since it caused a decrease in total cholesterol (16.9%), glycosylated hemoglobin (22.7%), fasting glucose (32.2%), LDL cholesterol (35.6%), and triglyceride (56.4%) levels, while increasing HDL cholesterol levels (27.6%). Treatment with *S. officinalis* was well tolerated since no adverse effects were reported [69].

Another work from the same year [70], a randomized, double-masked, and placebo-controlled study with 80 type II diabetic patients, also aimed to evaluate the antihyperglycemic and lipid profile-improving properties of *S. officinalis* extract. Briefly, intake of 150 mg of *S. officinalis* extract three times a day in addition to routine antidiabetic treatment caused a significant reduction in total cholesterol and two hours of postprandial glucose levels. Nonetheless, fasting glucose, glycosylated hemoglobin, and other lipid parameters were not affected when compared with the placebo group after 3 months off treatment [70]. Unfortunately, the authors do not clarify which type of extract it is, nor which supplier, which significantly reduces the impact of the obtained results, since there is no possibility of proceeding to later stages or comparing with other published studies.

*Salvia officinalis* extract was also used in another randomized, double-masked, parallel-group, placebo-controlled trial to ascertain its synergistic effect with routine diabetes and cholesterol treatments [71]. After randomization of the 105 hypercholesterolemic type II diabetic patients into an extract group or a placebo group, in addition to their daily use of 10 mg of atorvastatin, 15 mg of glyburide, and 2000 mg metformin, patients were instructed to take every 8 h one extract capsule (500 mg of *S. officinalis* hydroethanolic extract powder) or placebo capsule (500 mg of toast powder) for 2 months. Compared with the placebo group, HDL cholesterol levels increased by 11.23% while glycosylated hemoglobin (6.94%), fasting glucose (10.97%), 2 h postprandial glucose (23.53%), LDL cholesterol (6.74%), total cholesterol (19.98%), and triglyceride (34.66%) levels decreased in the extract group at the end of treatment. Throughout the trial period, no adverse drug reactions were reported, thus demonstrating that supplementation with *S. officinalis* hydroethanolic extract to improve routine diabetes and cholesterol treatments is well tolerated [71].

*Salvia miltiorrhiza* is another species frequently used to manage diabetes, resulting in increased attention recently, including a protocol for a meta-analysis and systematic review to analyze the various published pre-clinical and clinical trials that use *S. miltiorrhiza* [72]. In fact, one review and two meta-analyses and systematic reviews have been carried out to scrutinize the published works that stated the *S. miltiorrhiza* antidiabetic properties, either alone [73] or in conjugation with other medicinal plants widely used in Chinese traditional medicine, i.e., *Ligusticum striatum* DC. [74] or *Panax notoginseng* (Burkill) F.H.Chen ex C.Y.Wu and K.M.Feng [75]. The overall main conclusion of the mentioned reviews is that *S. miltiorrhiza* can be seen as a safe and efficient new approach for the prevention and handling of diabetes since it regularly and significantly regulates serum levels of glycosylated hemoglobin and fasting blood glucose in diabetics; however, all the different authors argue that, due to poor conceptualized methodology and other limitations of some included studies, further scientific evidence is still required from larger randomized clinical trials with higher-quality design [73,74,75].

#### 2.2.2. Cardiovascular Health

Cardiovascular health is a very general term that refers to the overall state of the heart, blood vessels, and blood parameters (e.g., glucose and lipids levels), the occurrence of a great range of disorders (e.g., atherosclerosis, hypertension, and stroke), and development of numerous co-morbidities, such as dyslipidemia, hypertension, and/or obesity [76]. The use of therapeutic plant mixtures to combat cardiovascular problems is a habitual approach, particularly in Asia [77]. Consequently, various clinical trials aiming to assess the efficacy and clinical safety of *Salvia* species treatment were found.

1. Angina

Angina can be described as a constricting discomfort or pain that characteristically occurs in the chest and is triggered by emotional stress or physical effort [78]. Improvements in life quality by controlling angina symptoms can be achieved through nitrate salts medication; however, the development of tolerance is common [79]. Thus, the search for alternative therapies is necessary.

*Salvia miltiorrhiza* depside salt (SMDS) is an emerging drug composed of the water-soluble purified compounds from *S. miltiorrhiza* (i.e., mainly rosmarinic acid, lithospermic acid, and magnesium lithospermate B) that received a lot of attention in the last years because of its role of promoting blood circulation [80]. In fact, a systematic review and meta-analysis aiming to clarify the clinical safety and efficiency of SMDS was published in 2017 [81]. The results of 5503 patients from 56 randomized controlled clinical trials were analyzed, and despite the frequently reported side effects of dizziness and palpitation, the conclusion is that the SMDS, when combined with clinically approved medication, can provide good outcomes for patients with coronary heart disease, particularly the ones with angina symptoms; however, it is warned by the authors to take caution when interpreting this conclusion, since the majority of the analyzed clinical trials presented poor methodological quality and lack of rigor in their discussions.

Furthermore, in the same year, another study [82] investigated safety issues regarding the use of SMDS in a clinical scenario, i.e., hospitals. Data from 30,180 patients prescribed with SMDS in 36 hospitals was analyzed, and it was determined that treatment with SMDS was well accepted by patients, with the incidence of adverse effects (drug reactions, drug events, and events) being, respectively, 0.79%, 1.57%, and 6.40%.

More recently, Lyu et al. [83] assessed the clinical effectiveness of using SMDS, in combination with aspirin, to treat stable angina, pectoris patients. A total of 135 participants were randomized into three groups, i.e., the control group (0.1 g/day of aspirin; n = 43), the SMDS group (0.2 g/day; n = 43), and the combination group (0.2 g/day of SMDS + 0.1 g/day of aspirin; n = 49). They were treated with the corresponding substances for 10 days. Afterward, the only statistically significant outcome was the one observed in the combination treatment, namely the highest enhancement of platelet inhibition rate induced by the arachidonic acid pathway, which increased from 30.6% at baseline to 81.6% after treatment. Unfortunately, the authors present the rest of the obtained results in the form of graphics without specifying the correct values for each group, thus impairing an accurate comparison between them.

A meta-analysis and systematic review [84] aimed to gauge the security and efficiency of natural compounds critically pellet and a nitrate salt medication, which have become widely used in China to treat angina, i.e., natural drug including *Salvia* compounds, when compared with conventional drug treatment, i.e., nitrate salt medication. Derived from traditional Chinese medicine, this natural drug is defined as a mixture of active compounds extracted from *Cinnamomum camphora* (L.) J. Presl, *P. notoginseng*, and *Salvia miltiorrhiza* Bunge. The results of 51 randomized controlled clinical trials (a total of 4732 patients diagnosed with chronic stable angina) were scrutinized with the intention of finding credible proof for the clinical practice of the drug. While admitting some limitations in their study (e.g., the inclusion of results from poor methodological designed trials), the authors conclude that when compared with nitrates salt medication, a natural compound pellet can be more effective with fewer adverse events in ameliorating angina symptoms, however, one must take into account that the effects detected may be due to the effect of the other two plants included in the formulation of natural drug, or to a synergistic effect of two or more of those components.

2. Coronary Heart Disease

Also known as coronary artery disease, coronary heart disease is a cardiovascular disorder characterized by impaired endothelial function of the arterial wall and its consequent disruption, which will lead to lipoprotein content accumulation in the endothelium of coronary vessels, increasing the risk of myocardial infarction, stable or unstable angina, and/or sudden cardiac death events [85].

There are some reports of clinical evidence on the effect of pure compounds extracted from *Salvia* species on coronary heart disease, the most promising one being sodium tanshinone IIA sulfate [86]. Derived from *S. miltiorrhiza* roots, this powerful anti-inflammatory compound was recently well reviewed [87], being addressed in various clinical trials that attest to its cardiovascular-improving properties. For instance, a placebo-controlled, single-center, randomized clinical trial discovered that the use of sodium tanshinone IIA sulfate (60 mg/day for 10 days) with thrombolysis therapy in 42 acute ischemic stroke patients contributed to their elevated neurologic functional outcomes by diminishing blood-brain barrier disruption [88].

Continuing with adjunctive treatments, Shang and colleagues [89] published a protocol for a clinical trial aiming to compare 72 patients with coronary heart disease who, for 14 days, would receive 20 mg/day of simvastatin (control, n = 36), with another group of patients receiving an additional 80 mg/day of sodium tanshinone IIA sulfate (n = 36). The authors expected that the combined therapy would show a synergistic effect, reducing circulating inflammatory markers and improving blood stasis syndrome and angina symptoms in participating patients. Although no evidence was found for the application of this protocol in a clinical trial, it demonstrates an interest in exploring the potential synergistic effects of *Salvia* species with therapeutic medicines in the treatment of coronary diseases.

Liu and colleagues [90] conducted a prospective, placebo-controlled, and randomized clinical trial aiming to investigate whether a pill of a mixture of compounds derived from the ethanolic extract of *S. miltiorrhiza* could reduce coronary heart disease aggravation risk in 126 patients. Each pill contains 27 mg of starch plus the majority of compounds present in *S. miltiorrhiza* ethanolic extract (i.e., 0.04% of tanshinone I, 0.21% of cryptotanshinone, 0.28% of tanshinone IIA, 1.2% of rosmarinic acid, and 5.8% of salvianolic acid B). After randomization, each patient took three daily pills of this mixture or three placebo pills, according to their group. After three months of trial, when compared with the placebo group, the compounds mixture treatment group reduced the levels of direct bilirubin, gamma-glutamyl transpeptidase, homocysteine, lipoprotein (a), LDL cholesterol, total cholesterol, triglycerides, and uric acid (*p* < 0.05). In contrast, levels of HDL cholesterol, indirect bilirubin, total bilirubin, and apolipoprotein A, B, and E were increased in the compound’s mixture treatment group (*p* < 0.05). Based on the obtained results, the authors conclude that the *S. miltiorrhiza* pill can be used to reduce coronary heart disease risk through improvement of individual lipid profile, as well as their liver and renal functions [90].

Several clinical studies with *Salvia* extracts or mixtures of these and other medicinal plants were carried out to assess their effects on coronary artery disease. For instance, a systematic review and meta-analysis were carried out to clarify if *S. miltiorrhiza* extract was better at treating cerebral infarction than a mixture of *Carthamus tinctorius* L. and *S. miltiorrhiza* extract. The analysis of 12 randomized controlled trials involving 1044 patients who suffered some degree of cerebral infarction revealed that the mixture extract was statistically significantly better than the *S. miltiorrhiza* extract alone. In fact, when combined with routine therapies, the mixture extract was 27% superior to just *S. miltiorrhiza* extract in improving clinical outcomes [91].

A pilot double-masked placebo-control study where 50 of 100 patients with coronary heart disease took capsules containing a mixture powder of aqueous extracts from *S. miltiorrhiza* and *Pueraria montana* var. *lobata* (Willd.) Maesen and S.M.Almeida ex Sanjappa (syn. *Pueraria lobata* (Willd.) Ohwi) (3 g/day) for 24 weeks. After the combinatory treatment, no significant adverse events were reported, and when compared with the placebo group, the carotid intima-media thickness and the brachial arterial endothelial function saw significant improvements (*p* < 0.05) [92]. In addition, for this study, the impact of the obtained results is strongly diminished by the fact that little is known about the composition of the tested mixture. For example, whether it is a commercial mixture or prepared by the authors and in what proportion the species extracts are combined.

*Salvia miltiorrhiza* and *P. montana* var. *lobata* mixture were also used in another double-masked, placebo-controlled, parallel, and randomized study. After randomization and for 12 months, 90 patients with high-risk hypertension were prescribed with placebo or doses of 1 g/day or 2 g/day. The results point out that the only parameters that improved significantly were the brachial flow-mediated dilation (increased by 32.2%; *p* < 0.0001) and the carotid intima-media thickness (decrease of 4.1%; *p* < 0.0001), with the higher dose group presenting the best results. In comparison, the placebo group presented an increase of only 15.5% (*p* = 0.028) for brachial flow-mediated dilation and, in contrast, an increase of 1.3% (*p* = 0.510) of the carotid intima-media thickness [93].

A large double-masked, multi-center, parallel controlled, randomized clinical trial aimed to evaluate the effectiveness and safety of another emerging drug in Chinese medicine, named Qi-Shen-Yi-Qi Dripping Pills (QSYQ), regarding its role as secondary prevention of myocardial infarction [94]. QSYQ is a mixture of extracts from different plants known for their medicinal properties, i.e., *Astragalus mongholicus* Bunge, *Dalbergia odorifera* T.C.Chen, *P. notoginseng*, and *S. miltiorrhiza*. A total of 3505 individuals who had previously suffered at least one diagnosticated myocardial infarction were recruited and randomly assigned to the QSYQ group (1.5 g/day) or the aspirin group (0.1 g/day). After 12 months, the treatment ended, and a follow-up visit took place six months later, being noted during all this period the number of occurrences of serious vascular events. The rate of vascular events incidence (i.e., non-fatal myocardial infarction, non-fatal stroke, and cardiovascular death) at 12 months and 18 months were, respectively, 2.96% and 3.81% in the aspirin group and 2.98% and 3.67% in the QSYQ group. Since no significant variances were detected between treatments, the authors of the study [94] suggest that QSYQ can be used as an alternative supplementary therapy for secondary prevention of myocardial infarction since it showed similar prevention effects to aspirin. It is worth noting that this study does not include a placebo group, which limits its conclusions by increasing the risk of bias, making the treatment received more evident, and not evaluating the absolute effectiveness of the drug being tested.

3. Dyslipidemia

Dyslipidemias are normally characterized by atypical blood serum levels of cholesterol and/or triglycerides and of the lipoprotein types in which they are transported, i.e., HDL, LDL, and very-low-density lipoproteins (VLDL) [95]. Its prevalence can result in a higher risk of developing atherosclerotic cardiovascular diseases (e.g., stroke); thus, controlling cholesterol and/or triglycerides to stay at normal levels is imperative [96].

Kianbakht and colleagues [97] carried out a double-masked, placebo-controlled, randomized clinical study with 67 hyperlipidemic subjects that, for 2 months, taken every 8 h, a 500 mg capsule containing *S. officinalis* hydroethanolic leaf extract powder or placebo (toast powder). After the treatment period, when compared with the placebo group, the lipid profile of the subjects in the extract group suffered a significant change. Blood levels of VLDL, LDL, total cholesterol, and triglyceride suffered reductions of 13.3%, 19.7%, 19.6%, and 22.8%, respectively. In contrast, blood levels of HDL increased by 20.2%. Even though no adverse effects were reported and antihyperlipidemic effects of *S. officinalis* were observed, the authors suggest that additional clinical trials addressing the effectiveness and safety of treatment should take place before widespread use of the extract [97]. It should be noted that in clinical studies involving extracts, as in a previous study, it is common not to find data relating to the extract used. For example, who is the supplier, what is the proportion between the various extracts, what is the chemical composition of the extract or mixture, or whether it is standardized. This, after the lack of a placebo group, is one of the methodological aspects that most weakens the impact of the obtained results.

*Salvia officinalis* aqueous extract was also used in a very small, non-randomized crossover pilot trial where six healthy female volunteers consumed, two times a day, 300 mL of a lyophilized infusion solution (3.5 ± 0.1 mg/mL), for four weeks, in order to study the possible advantageous characteristics of the plant on transaminase activity, lipid profile and blood glucose regulation of the subjects. To establish baseline values for all the subjects, a two-week baseline period was implemented, in addition to a two-week wash-out phase, to evaluate the prevalence of effects past the treatment period. At the end of the trial, the results indicated that drinking *S. officinalis* infusion solution had no effects on plasma glucose levels but reduced plasma total cholesterol levels by 16%, as well as LDL levels by 19.6%. In addition, HDL levels saw a 50.6% increase at the end of the treatment and were still at 37.6% higher than baseline after the two-week wash-out period [98]; however, the results of this work should not be taken as robust data since a placebo group was not included, and the number of subjects was very small.

*Chia* seeds (*S. hispanica*) are a widely used dietary supplement, primarily taken for their high content of α-linolenic acid, dietary fiber, and plant-based protein [99]. Given its ever-increasing presence in human diets, several clinical trials with different outcomes have been carried out to assess its role in human health and provide scientific evidence for its consumption [100,101]. Regarding the effect of *S. hispanica* on cardiovascular health, a systematic review from 2015 [102] examined seven clinical trials and concluded that, because of the poor methodology in the majority of the trials, there was no notable statistical connection between alteration of cardiovascular risk factors and *S. hispanica* seed consumption. More recently, Silva and colleagues [103] published a systematic review and meta-analysis involving 10 clinical trials and data from 489 participants. The results point out that *S. hispanica* consumption, in addition to improving the lipid profile by elevating HDL levels and lowering LDL, total cholesterol, and triglycerides levels, can lead to an increase in the serum levels of α-linolenic acid, eicosapentaenoic acid, and other ω-3 fatty acids. Despite the optimistic outcomes presented, the authors warn that to obtain undisputable proof of the positive impact of *S. hispanica* consumption, further clinical trials are needed, with better methodological quality, longer interventions, and larger samples.

In addition to *S. officinalis* and *S. hispanica* extracts, *S. miltiorrhiza* extract was also used in clinical trials aiming to attest to their capacity to ameliorate cardiovascular health; however, these are trials in which the species is used in combination with other plants.

Capsules containing a mixture powder of aqueous extracts from *S. miltiorrhiza* and *P. montana* var. *lobata* were taken (1 g/day) by 165 postmenopausal women with early hyper-cholesterolemia in a double-masked, randomized, placebo-controlled trial for 12 months. After the treatment period, the subjects exhibited significant (*p* < 0.004) reductions in the serum levels of LDL cholesterol (6.92%), total cholesterol (5.85%), and the intima-media thickness of their carotid artery (1.52%), while a decrease in the placebo group (*p* = 0.009) was 3.21%, 3.42%, and 1.13%, respectively [104]. Unfortunately, in addition to saying that “the study herbal product was produced by a good manufacturing practice qualified Chinese Herbal Medicine manufacturer” (without indicating where or who this manufacturer is), the authors never state the composition (e.g., the proportion of each plant) of the tested capsules which impairs the strength of the study since it makes it impossible to replicate and/or compare with other studies.

A synergistic effect of combining different medicinal products with *S. miltiorrhiza* was reported at a 12-week parallel double-masked randomized placebo-controlled with 40 cholesterolemic participants [105]. The study found that the blood levels of the adhesion molecules surface proteins E-selectin and ICAM-1 (risk factors for aggravation of cardiovascular disease) were decreased by the Dantonic pill, a Chinese medicine containing borneolum syntheticum and an herbal mixture of *S. miltiorrhiza* and *P. notoginseng.* In fact, the treatment led to a statistically significant reduction in ICAM-1 (by 9%; *p* = 0.03) and E-selectin (by 15%; *p* = 0.004) levels. Although there were reductions in the placebo group, they were not statistically significant [105].

**Table 3:** Summary of clinical studies and their results involving *Salvia* species.

Disease	*Artemisia* Species	Compound/Drug/Extract	Type of Study	Number of Participants	Intervention	Main Outcomes	Reference
Diabetes	*Salvia officinalis* L.	Extract (Ethanolic)	Randomized, Placebo-controlled, and Triple-blinded Clinical Trial	70 (Polycystic ovary syndrome)	2 Treatments (8 weeks): Placebo Group (n = 35) = 300 mg/day of Corn starch, orally 1 capsule Extract Group (n = 35) = 300 mg/day of Extract, orally 1 capsule	Body mass index: Extract Group = Significant (*p* = 0.001) ↓ by 0.56 ± 0.90 kg/m^2^ Placebo Group = ↑ by 0.08 ± 0.38 kg/m^2^ Insulin levels: Extract Group = Significant (*p* < 0.001) ↓ by 28.03% Placebo Group = ↓ by 1.68%	[68]
		Extract (Hydroethanolic leaf extract)	Randomized, Placebo-controlled, and Parallel group Clinical Trial	80 (Hyperlipidemic Type II diabetic)	2 Treatments (3 months): Placebo Group (n = 40) = 500 mg/8 h of Toast powder, orally 1 capsule + 10 mg/day of Glyburide, orally 2 capsules (5 mg each) + 1000 mg/day of Metformin, orally 2 capsules (500 mg each) Extract Group (n = 40) = 500 mg/8 h of Extract, orally 1 capsule + 10 mg/day of Glyburide, orally 2 capsules (5 mg each) + 1000 mg/day of Metformin, orally 2 capsules (500 mg each)	Antihyperglycemic and lipid profile improving effects in Extract Group: ↓ Total cholesterol by 16.9% (*p* < 0.01) ↓ Glycosylated hemoglobin by 22.7% (*p* < 0.01) ↓ Fasting glucose by 32.2% (*p* < 0.001) ↓ LDL cholesterol by 35.6%) (*p* < 0.001) ↓ Triglyceride by 56.4%) (*p* < 0.001) ↑ HDL cholesterol by 27.6% (*p* < 0.008)	[69]
		Extract (Not specified)	Randomized, Placebo-controlled, and Double-masked Clinical Trial	80 (Type II diabetic)	2 Treatments (3 months): Placebo Group (n = 40) = Placebo (not specified), orally 3 capsules/day Extract Group (n = 40) = 450 mg/day of Extract, orally 3 capsules (150 mg each)	Significant (*p* < 0.05) ↓ in 2 h postprandial glucose and total cholesterol levels in Extract Group	[70]
		Extract (Hydroethanolic)	Randomized, Double-masked, and Placebo-controlled Clinical Trial	105 (Hypercholesterolemic type II diabetic)	2 Treatments (2 months): Placebo Group (n = 53) = 500 mg/8 h of Toast powder, orally 1 capsule + 10 mg/day of Atorvastatin + 15 mg/day of Glyburide + 2000 mg/day of Metformin Extract Group (n = 52) = 500 mg/8 h of Extract, orally 1 capsule + 10 mg/day of Atorvastatin + 15 mg/day of Glyburide + 2000 mg/day of Metformin	Antihyperglycemic and lipid profile improving effects in Extract Group: ↓ Glycosylated hemoglobin by 6.94% ↓ Fasting glucose by 10.97% ↓ 2 h postprandial glucose by 23.53% ↓ Total cholesterol by 19.98% ↓ LDL cholesterol by 6.74% ↓ Triglyceride by 34.66% ↑ HDL cholesterol by 11.23%	[71]
Angina	*Salvia miltiorrhiza* Bunge	SMDS ^1^	Multi-center, Pragmatic, Randomized, and Controlled Clinical Trial	135 (Stable angina pectoris)	3 Treatments (10 days): Control Group (n = 43) = 100 mg/day of Aspirin, orally 1 pill 1×/day SMDS Group (n = 43) = 200 mg/day of SMDS, intravenous drip 1×/day Combination Group (n = 49) = 100 mg/day of Aspirin, orally 1 pill 1×/day + 200 mg/day of SMDS, intravenous drip 1×/day	Significant (*p* < 0.001) ↑ in platelet inhibition rate induced by the arachidonic acid pathway in Combination Group, from 30.6% to 81.6%	[83]
Coronary Heart Disease	*Salvia miltiorrhiza* Bunge	STS ^2^	Placebo-controlled, Single-center and Randomized Clinical Trial	42 (Acute ischemic stroke)	2 Treatments (10 days): Placebo Group (n = 21) = Saline (equivalent volume of STS), intravenously administrated 1×/day STS Group (n = 21) = 60 mg/day of STS, intravenously administrated 1×/day	↑ Neurologic function in STS Group by diminishing blood-brain barrier disruption	[88]
		Danshen ^3^	Prospective, Placebo-controlled, and Randomized Clinical Trial	126 (Coronary heart disease)	2 Treatments (3 months): Placebo Group (n = 63) = Placebo (not specified), orally 1 pill 3×/day Danshen Group (n = 63) = 81 mg/day of Danshen, orally 1 pill (27 mg) 3×/day	Significant (*p* < 0.05) ↓ coronary heart disease aggravation risk in the Danshen Group: ↓ Direct bilirubin ↓ Gamma-glutamyl transpeptidase ↓ Homocysteine ↓ Lipoprotein (a) ↓ LDL cholesterol ↓ Total cholesterol ↓ Triglycerides ↓ Uric acid ↑ HDL cholesterol ↑ Indirect bilirubin ↑ Total bilirubin ↑ Apolipoprotein A, B, and E	[90]
		Extracts Mixture (Mixture powder of aqueous extracts from *S. miltiorrhiza* and *Pueraria montana* var. *lobata* (Willd.) Maesen and S.M.Almeida ex Sanjappa (syn. *Pueraria lobata* (Willd.) Ohwi)	Pilot, Double-masked, and Placebo-control Clinical Trial	100 (Coronary heart disease)	2 Treatments (3 months): Placebo Group (n = 50) = Placebo (not specified), orally 1 capsule 6×/day Mixture Group (n = 50) = 3000 mg/day of Mixture powder, orally 1 capsule (500 mg) 6×/day	Significant (*p* < 0.05) ↑ carotid intima-media thickness in Mixture Group Significant (*p* < 0.05) ↑ brachial arterial endothelial function in Mixture Group	[92]
		Herbal Drug (Mixture powder of *S. miltiorrhiza* and *P. montana* var. *lobata* dried roots)	Parallel, Placebo-controlled, Randomized and Double-masked Clinical Trial	90 (High-risk hypertension)	3 Treatments (12 months): Placebo Group (n = 29) = Placebo (not specified), orally 1 capsule/day Low-dose Group (n = 31) = 1000 mg/day of Mixture powder, orally 1 capsule/day High-dose Group (n = 30) = 2000 mg/day of Mixture powder, orally 1 capsule/day	Brachial flow-mediated dilation: ↑ in Placebo Group by 15.5% (*p* = 0.028) ↑ in Low-dose Group by 27.8% (*p* < 0.0001) ↑ in High-dose Group by 32.2% (*p* < 0.0001) Carotid intima-media thickness: ↑ in Placebo Group by 1.3% (*p* = 0.510) ↓ in Low-dose Group by 3.4% (*p* = 0.001) ↓ in High-dose Group by 4.1% (*p* < 0.0001)	[93]
		QSYQ ^4^	Double-masked, Multi-center, Parallel Controlled and Randomized Clinical Trial	3505 (Myocardial infarction)	2 Treatments (12 months + Follow-up 6 months after): Aspirin Group (n = 1759) = 1500 mg/day of Simulated QSYQ, orally 1 package (500 mg) 3×/day + 100 mg/day Aspirin, orally 4 pills 1×/day QSYQ Group (n = 1746) = 1500 mg/day of QSYQ, orally 1 package (500 mg) 3×/day + 100 mg/day Simulated Aspirin, orally 4 pills 1×/day	Rate of vascular events incidence at 12 months: Aspirin Group = 2.96% QSYQ Group = 2.98% Rate of vascular events incidence at 18 months: Aspirin Group = 3.81% QSYQ Group = 3.67%	[94]
Dyslipidemia	*Salvia miltiorrhiza* Bunge	Extracts Mixture (Mixture powder of aqueous extracts from *S. miltiorrhiza* and *P. montana* var. *lobata*)	Double-masked, Randomized and Placebo-controlled Clinical Trial	165 (Postmenopausal with early hyper-cholesterolemia)	2 Treatments (12 months): Placebo Group (n = 80) = Placebo (not specified), orally 1 capsule 2×/day Mixture Group (n = 85) = 1000 mg/day of Mixture powder, orally 1 capsule (500 mg) 2×/day	Significant (*p* < 0.004) ↓ in Mixture Group: LDL (6.92%) Total cholesterol (5.85%) Carotid intima-media thickness (1.52%) ↓ in Placebo Group (*p* = 0.009 LDL (3.21%) Total cholesterol (3.42%) Carotid intima-media thickness (1.13%)	[104]
		Dantonic ^5^	Parallel, Double-masked, Randomized and Placebo-controlled Clinical Trial	40 (Cholesterolemic)	2 Treatments (12 weeks): Placebo Group (n = 20) = Placebo (not specified), orally 3 capsules 3×/day Dantonic Group (n = 20) = Dantonic (not specified concentration), orally 3 capsules 3×/day	Significant ↓ in adhesion molecules surface proteins in the Dantonic Group: ↓ ICAM-1 levels by 9% (*p* = 0.03) ↓ E-selectin levels by 15% (*p* = 0.004)	[105]
	*Salvia officinalis* L.	Extract (Hydroethanolic leaf extract)	Double-masked, Placebo-controlled, and Randomized Clinical Trial	67 (Hyperlipidemic)	2 Treatments (2 months): Placebo Group (n = 33) = 500 mg/8 h of Toast powder, orally 1 capsule Extract Group (n = 34) = 500 mg/8 h of Extract, orally 1 capsule	Lipid profile improved in Extract Group: ↓ VLDL by 13.3% (*p* = 0.002) ↓ LDL by 19.7% (*p* = 0.143) ↓ Total cholesterol by 19.6% (*p* < 0.001) ↓ Triglyceride by 22.8% (*p* < 0.001) ↑ HDL by 20.2% (*p* = 0.001)	[97]
		Extract (Aqueous)	Non-randomized, Crossover, and Pilot Clinical Trial	6 (Healthy)	Treatment (2 Weeks Baseline + 4 Weeks + 2 Weeks Wash-out): 300 mL of a lyophilized infusion solution (3.5 ± 0.1 mg/mL), orally 2×/day	↓ Total cholesterol by 16% ↓ LDL by 19.6% ↑ HDL by 50.6% HDL 37.6% higher than baseline after the two-week wash-out period	[98]

^1^ SMDS = *Salvia miltiorrhiza* depside salt; ^2^ STS = Sodium tanshinone IIA sulfate; ^3^ Danshen = 27 mg pill composed of starch and compounds derived from *S. miltiorrhiza* ethanolic extract (i.e., 0.28% tanshinone IIA, 0.21% cryptotanshinone, 0.04% tanshinone I, 1.2% rosmarinic acid, 5.8% salvianolic acid B); ^4^ QSYQ = Qi-Shen-Yi-Qi dripping pill is a mixture of extracts from different plants known for their medicinal properties, i.e., *Astragalus mongholicus* Bunge, *Dalbergia odorifera* T.C.Chen, *P. notoginseng* and *S. miltiorrhiza*; ^5^ Dantonic = A Chinese medicinal pill containing borneolum syntheticum and an herbal mixture of *S. miltiorrhiza* and *P. notoginseng*; ↑ = Higher or increase; ↓ = Lower or decrease; HDL = High-density lipoprotein; LDL = Low-density lipoprotein; VLDL = Very-low-density lipoprotein.

## 3. Clinical Trials Overview

Regarding clinical trials involving *Artemisia*, there are more studies focused on its effects on diabetes, but research into its anticancer efficacy is more advanced, as it involves some of its pure compounds, with artesunate being the most frequently tested compound among them. Concerning *Salvia*-related clinical trials, studies aiming to improve cardiovascular health were predominant.

*Salvia miltiorrhiza* was by far the most commonly targeted plant in the various clinical trials stated above, either isolated compounds or extracts, alone or in conjunction with other medicinal plants, which is not surprising given its history of broad cardiovascular protective properties [106,107].

Clinical trials using mixtures (dried powdered plants or extracts) of known medicinal plants are recurrent in published works, in contrast with the, unfortunately, scarce clinical trial studies with isolated compounds. Several factors can be pointed out to justify the lack of clinical trials using pure compounds, the main one being the inability to obtain active pure compounds in sufficient quantities to carry out the trials.

The findings from the various clinical trials mentioned above offer clinical proof to back up the use of plants from *Artemisia* and *Salvia* genera as sources of extracts and compounds to treat cancer, diabetes, and cardiovascular diseases. Some of these extracts and compounds are in advance stages of study or, particularly in China, being already well-established and accepted medicines.

Overall, both plant genera have tremendous potential to offer new solutions in the treatment of several diseases; however, further higher-quality scientific studies need to take place with bigger randomized clinical trials to present a strong backup to the already available information.

As noted throughout the text, a substantial portion of the studies reviewed demonstrate low methodological quality. Some of them have already been acknowledged by the authors themselves and/or recognized by other authors in their systematic reviews. Unfortunately, several studies stated here were conducted with very small population samples, which limits their statistical power. Another major flaw is the poor conceptualized methodology, similar to not including a placebo group in the study or not specifying the composition of the placebo used, not providing concrete data regarding the characterization of the extracts used in the clinical studies, or not stating the scientific name of the plant used. Additionally, it should be noted that clinical studies using only one dose of extract limit the understanding of the dose-response relationship, not allowing the identification of a possible therapeutic window.

## 4. Conclusions and Perspectives

Taking into account all the information stated above, it is notorious that, although not all perfectly designed and executed, the clinical trial results demonstrate the tremendous potential of *Artemisia* and *Salvia*.

In terms of cancer, *A. annua* offers various options, i.e., artemisinin and its analogs, to develop more studies and drugs against cancer since it has been demonstrated to be effective in decreasing tumor mass and slowing their proliferation.

*Artemisia* and *Salvia* genera can provide various options in terms of diabetes treatment, with *A. absinthium* standing out in lowering and maintaining fasting serum glucose levels with only a 30-day treatment.

*Salvia miltiorrhiza* can be highlighted as the best plant to improve coronary heart disease symptoms since it affects various parameters, i.e., lowering lipoprotein (a), LDL cholesterol, total cholesterol, and triglycerides while increasing HDL cholesterol.

This review presented here underscores an important reality: while numerous trials have been conducted, there is limited clinical evidence with statistical significance and robust and reproducible results. Consequently, many of the reported findings lack meaningful impact and fall short of advancing the introduction of new drugs into clinical practice. This situation calls for a higher standard of rigor and accountability from researchers and regulatory agencies to enhance the quality and reliability of clinical and case studies being conducted.

Future research should prioritize high-quality clinical trials to validate the therapeutic potential of *Artemisia* and *Salvia*. *A. annua* shows promise in cancer treatment, while *Salvia miltiorrhiza* stands out for cardiovascular health. Rigorous methodologies and standardized studies are essential to ensure clinical translation. Journal reviewers and editors must be very demanding and careful when reviewing and accepting manuscripts with case and clinical study results in order to publish only quality data.

## Figures and Tables

**Table 1:** Completed clinical trials listed on ClinicalTrials.gov that are *Artemisia* and *Salvia*-related and relevant to the theme of this review.

Plant Species	Title	NCT Number
*Artemisia absinthium* L.	Safety and Efficacy of Herbal Tea in Type 2 Diabetics	NCT04054284
	NUTRACORE, Glycaemic Index and Appetite	NCT05528874
*Artemisia annua* L.	Study of Artesunate in Metastatic Breast Cancer	NCT00764036
*Artemisia argyi* H.Lév. and Vaniot	Clinical Trial to Evaluate the Efficacy and Safety of CKD-495 Tablet	NCT03437785
	A Clinical Trial to Evaluate the Efficacy and Safety of CKD-495	NCT04255589
*Artemisia capillaris* Thunb.	The Efficacy of Tradition Chinese Medicine in Patients with Irritable Bowel Syndrome	NCT00676975
*Artemisia dracunculus* L.	Safety and Efficacy Study of Tarragon on Insulin Action in Humans	NCT01057576
	Evaluation of Soy-protein Russian Tarragon (*Artemisia Dracunculus* L.)	NCT02088203
	Effect of Artemisia Dracunculus on Glucose Intolerance, Insulin Sensitivity and Insulin Secretion	NCT02330341
*Salvia hispanica* L.	Effect of Chia Seeds (*Salvia Hispanica* L.) on Glucose Control in Patients with Type 2 Diabetes	NCT00362011
	The Effects of *Salvia Hispanica*—Enriched Foods on Glycemic and Insulinemic Responses and Subjective Satiety	NCT00728065
	Effectiveness and Safety of Salba on Weight Loss in Overweight Individuals with Type 2 Diabetes	NCT01403571
	Effect of Salba & Flax on Postprandial Glycemia and Subjective Satiety	NCT02621307
	*Salvia Hispanica* Seed in Reducing Risk of Disease Recurrence in Patients with Non-Hodgkin Lymphoma	NCT02652715
	Chia Supplementation and Non-Alcoholic Fatty Liver Disease	NCT03942822
	Dose–Response Effect of Chia Seeds on Subjective Appetite and Glycemic Response	NCT05345470
*Salvia miltiorrhiza* Bunge.	Cardiovascular-Protective Effects of Herbal Medicine Danshen-Gegen	NCT01033630
	Danhong Injection in the Treatment of Acute Ischemic Stroke	NCT01677208
	Danhong Injection in the Treatment of Chronic Stable Angina	NCT01681316
	Dynamic Combination Therapy on Chinese Herbal Granules to Improve the Symptoms in Convalescent Phase of Ischemic Stroke	NCT01780480
	Danhong Injection in the Treatment of Unstable Angina Pectoris	NCT02007187
	Multi-“Omics” Research of Danhong Injection to Treat Acute Ischemic Stroke	NCT02176395
	An Intervention Study of Compound Silymarin in Patients with Non-alcoholic Fatty Liver Disease	NCT05497765
*Salvia officinalis* L.	Safety and Efficacy of Herbal Tea in Type 2 Diabetics	NCT04054284
